# The Visual N1 Is Sensitive to Deviations from Natural Texture Appearance

**DOI:** 10.1371/journal.pone.0136471

**Published:** 2015-09-10

**Authors:** Benjamin Balas, Catherine Conlin

**Affiliations:** 1 Department of Psychology, North Dakota State University, Fargo, ND, United States of America; 2 Center for Visual and Cognitive Neuroscience, North Dakota State University, Fargo, ND, United States of America; Ecole Polytechnique Federale de Lausanne, SWITZERLAND

## Abstract

Disruptions of natural texture appearance are known to negatively impact performance in texture discrimination tasks, for example, such that contrast-negated textures, synthetic textures, and textures depicting abstract art are processed less efficiently than natural textures. Presently, we examined how visual ERP responses (the P1 and the N1 in particular) were affected by violations of natural texture appearance. We presented participants with images depicting either natural textures or synthetic textures made from the original stimuli. Both stimulus types were additionally rendered either in positive or negative contrast. These appearance manipulations (negation and texture synthesis) preserve a range of low-level features, but also disrupt higher-order aspects of texture appearance. We recorded continuous EEG while participants completed a same/different image discrimination task using these images and measured both the P1 and N1 components over occipital recording sites. While the P1 exhibited no sensitivity to either contrast polarity or real/synthetic appearance, the N1 was sensitive to both deviations from natural appearance. Polarity reversal and synthetic appearance affected the N1 latency differently, however, suggesting a differential impact on processing. Our results suggest that stages of visual processing indexed by the P1 and N1 are sensitive to high-order statistical regularities in natural textures and also suggest that distinct violations of natural appearance impact neural responses differently.

## Introduction

Texture perception supports a range of important visual functions. Image segmentation [1] and material perception, for example, both rely to some degree on texture perception [2] as do some aspects of shape perception [3,4]. Recently, an even broader range of visual functions including visual search, the visual processing of crowded stimulus arrays in the periphery, and other tasks that are typically accomplished in the visual periphery have been successfully described vis-à-vis texture representations of appearance [5,6]. In general, texture perception and texture representations are described in terms of “summary statistics” that the visual system uses to describe the appearance of a texture. Though a range of feature vocabularies have been developed to account for performance in various tasks, texture representations typically are comprised of some set of image measurements that are aggregated over image regions such that location information is either greatly reduced or absent. Some examples of image descriptors that meet these criteria and have been applied to texture recognition problems of various types include dipole or “needle” statistics [7], moments of the pixel intensity histogram [8], “textons” that are derived from cluster analyses of natural images [9] multi-scale orientation histograms [10], and correlations between wavelets across position, orientation, and scale [11].

One useful way to characterize the neural code for texture appearance is to investigate how sensitive the visual system is to deviations from natural texture statistics. Visual processing is tuned to the statistics of natural images in a variety of ways. Computational analyses of natural scenes suggest that the receptive fields in early visual areas may reflect an optimal sparse code for representing and reconstructing natural inputs [12], for example. Spatial correlations between orientations in natural scenes also appear to be related to horizontal connections in early visual areas, suggesting that some joint orientation statistics are manifest in the visual system [13]. Behaviorally, there are many results that demonstrate how visual processing is adapted to the statistics of the natural visual world. The power spectrum of natural scenes tends to have a 1/f slope, for example [14] and human observers are better able to discriminate between noise patterns with different power-spectrum slopes if those slopes are near this value [15]. Considering higher-order statistics in natural textures, Balas [16] has demonstrated that observers are capable of distinguishing between real textures and synthetic textures that are constrained to match the original stimuli in terms of a wide range of joint wavelet statistics implemented by the Portilla-Simoncelli model [11] Moreover, the ability to distinguish between real and synthetic textures is modulated by contrast polarity: Observers can distinguish between positive contrast textures better than negative contrast textures, suggesting that pattern discrimination is tuned to the statistics of natural texture appearance [17]. This sensitivity to contrast polarity appears to be evident at 9 months of age [18] though other kinds of sensitivity to natural texture statistics are not evident until later in childhood [19]. Overall, these results (and others—see [20]) demonstrate that the human visual system is adapted to the statistics of natural images and understanding the limits of that sensitivity can reveal important clues about the underlying vocabulary for representation and recognition. Artificial textures, for example, can be matched for dipole statistics up to a very high order and still be discriminated [21], which likely means that other local statistics really drive performance in segmentation tasks. Similarly, if there are deviations from natural texture appearance that the visual system does not appear to be especially sensitive to, we can infer that visual features that reflect those deviations are likely not part of the feature vocabulary applied to those patterns. For example, Balas [16] demonstrated that images differing in cross-scale phase statistics were not discriminable in the periphery, from which it follows that these measurements are not available to the visual system. Characterizing the degree to which observers are sensitive to various deviations from natural texture appearance thus informs our understanding of the measurements the visual system uses to describe natural textures.

Recent results have used similar logic to examine the neural basis of texture processing, yielding important insights about where and how textures are processed by the visual system. Freeman & Simoncelli [22], for example, used synthetic textures like those employed by Balas [16,17] to determine the critical receptive field size at which they obtained “texture metamers” of natural scenes—texture-synthesized versions of complex images that were indistinguishable from the original. They found that receptive field sizes commensurate with cells in area V2 yielded metameric images, suggesting that texture codes for appearance are implemented at this level of the visual system. The sensitivity to differences between candidate metamers and natural scenes for other receptive field sizes suggests that there are deviations from natural appearance the visual system is sensitive to when synthetic images are rendered with synthesis parameters that are not consistent with the implementation of texture processing in the ventral stream. Using fMRI to study area V2 directly, Freeman et al. [23] also demonstrated that sensitivity to naturalistic texture appearance (which was operationalized using phase-scrambled versions of synthetic natural textures) was evident in V2, providing further evidence that computations similar to the summary statistics implemented in the Portilla-Simoncelli model may be carried out in this area. Again, we emphasize that sensitivity to the various differences between natural textures and texture images that deviate from natural appearance is a critically important tool for understanding both the vocabulary of texture processing and the neural basis of the same.

In the current study we chose to use visual event-related potentials (ERPs) as a tool for characterizing the visual system’s sensitivity to deviations from natural texture appearance. While a great deal of prior ERP work has largely been devoted to understanding texture processing in the context of segmentation tasks (often using artificial textures that vary according to the “textons” [24] that are present), recent results suggest that sensitivity to natural texture statistics may be evident at relatively early components of visual ERPs (though we point out that the term “early” is largely subjective and varies across reports). For example, natural textures and scenes tend to have contrast statistics that are well-described by a Weibull distribution with two free parameters [25] the width of the distribution of contrast values and the shape of the distribution. EEG responses measured over the visual cortex appear to largely be accounted for by variation in these properties, suggesting that visual processing of textures and scenes may be highly sensitive to regularities in the distribution of contrast energy in natural images [26]. Moreover, these same statistics also vary differently across viewing conditions and illumination changes as a function of texture category and this difference in the invariance of contrast statistics across ecologically relevant image transformation is also evident in visual ERPs [27]. Again, this result suggests that visual processing is sensitive to regularities in natural textures; in this case, specifically the distribution of unoriented contrast energy. We suggest that both these results and the aforementioned fMRI results suggest that the responses of visual areas such as V1 and V2 (and also other areas further along in the ventral stream) are tuned to natural texture statistics and that therefore, sensitivity to textures that deviate from the statistics of the natural world may also be evident at visual ERP components measured over the occipital cortex.

We compared the response of the P1 and N1 components to natural textures, synthetic textures created from the Portilla-Simoncelli model, and contrast-negated textures. Contrast-negation and texture synthesis are both useful ways to selectively disrupt a subset of natural statistics in images of real textures. Negation, for example, preserves the orientation energy and spatial layout of a given image, while reversing edge polarity globally. Contrast negation is known to disrupt face recognition substantially [28] and also to disrupt aspects of material perception [29] Texture synthesis, by comparison, constrains the subset of statistics used to describe texture appearance in the model under consideration (here the P-S model), but any features that are not included in the model description are unlikely to be matched between original textures and their synthetic counterparts. We selected these two transformations because they allow us to examine distinct aspects of the ERP response to natural vs. unnatural textures in the context of recent results describing how texture processing may proceed in visual cortex. As demonstrated by Freeman et al. [23] naturalistic structure as approximated by the P-S algorithm appears to be a reasonably good candidate vocabulary for describing area V2’s characteristics—to what extent then are differences between natural textures and the best approximations of natural textures made by the P-S algorithm available to the visual system? By examining when any differences between real and synthetic textures are evident, we will be able to determine if these are statistical differences the visual system encodes shortly after stimulus onset (and generalizes over in V2) or if these are differences that are primarily available later in the ERP waveform. Similarly, contrast negation allows us to specifically address the issue of whether sensitivity to natural vs. unnatural appearance is perhaps directly related to the contrast statistics that account for substantial EEG variability in Groen et al.’s work [27]. Though the wavelet correlations that are matched in the P-S algorithm largely preserve these statistics (meaning differences between natural and P-S textures are likely not driven by these parameters) contrast negation affords us the opportunity to examine this issue in a very direct way—since negation preserves both the contrast energy and the spatial layout of edges, both parameters of the underlying Weibull distribution describing contrast in a natural texture should be identical when edge polarity is reversed. These two transformations thus allow us to determine the sensitivity of visual ERP components to deviations from natural texture appearance while largely controlling for the contrast statistics that appear to explain a great deal of variance in EEG data recorded over occipital cortex.

We asked participants to carry out a same/different image discrimination task using our set of original and transformed textures to examine the following questions: (1) Do visual components (e.g. the P1 and N1) exhibit sensitivity to textures that differ from natural appearance? (2) Do responses to natural textures differ from response to unnatural textures in systematic ways, or do different transformations lead to differential effects at these components? Briefly, we found evidence of sensitivity to natural texture appearance. We also observed that contrast negation and texture synthesis lead to differential effects at the N1. We discuss these results in the context of prior behavioral work suggesting that the loss of information due to contrast negation and texture synthesis leads to different perceptual outcomes and also neural data demonstrating how texture appearance is represented in cortical visual areas.

## Methods

### Subjects

We recruited 17 participants (9 female) from the NDSU undergraduate community. All participants were between 18–26 years old and self-reported either normal or corrected-to-normal vision and were classified as right-handed according to the Edinburgh Handedness Inventory [30]. No participants reported any history of visual or neurological impairment. All participants gave written informed consent and all recruitment, consent and testing procedures were approved by the North Dakota State University IRB (protocol #SM11167).

### Stimuli

Our stimulus set was based on a set of 24 grey-scale images depicting a range of natural textures. These images were 512x512 pixels in size and included textures with a variety of material properties, such as fruits and vegetables, metal objects, wooden objects, and twine. To obtain multiple samples of these original textures (and their transformed versions that we describe below), we cropped a 256-pixel diameter circle from each quadrant of the image, yielding 4 unique texture patches from each original stimulus. The average pixel intensity for these images (range 0–255 gray values) was approximately 35.2 cd/m^2^.

**Fig 1:**
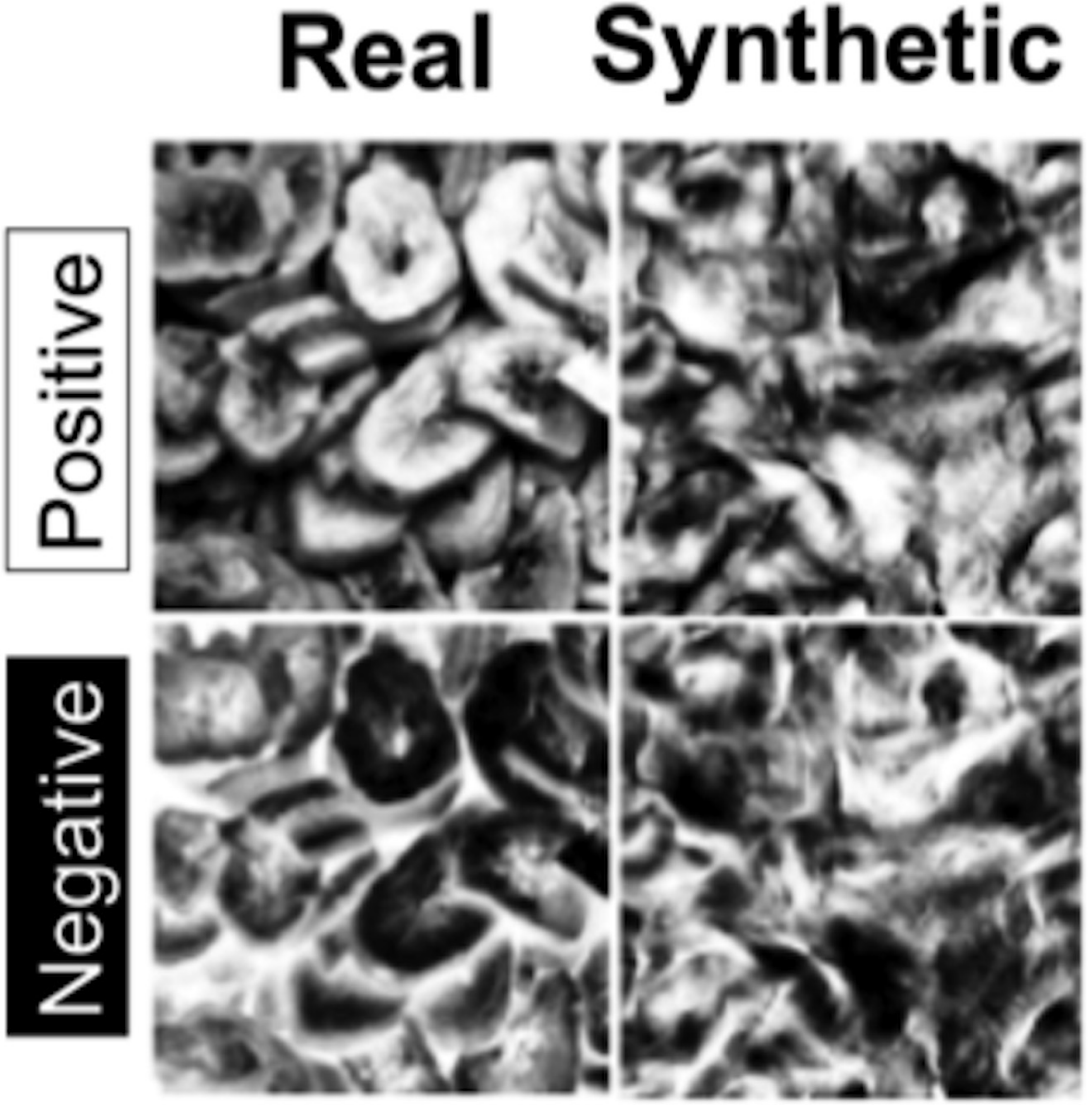
An example of a single original texture (upper left) and its synthetic counterpart (upper right), the original texture with contrast polarity reversed (lower left) and the result of applying both transformations (lower right).

To create the images we used in our ERP task, we implemented two transformations of natural appearance: contrast negation and texture synthesis. Contrast negation was implemented by simply reversing the intensity values of all pixels about the midpoint of the intensity scale for each image, yielding a new stimulus with black pixels exchanged for white, etc. based on a lookup table (this was done in the absence of any gamma correction). This procedure also ensured that pixel intensities were not saturated at either the low or high end of the intensity scale. Each original image was also used to generate a synthetic texture using the Portilla-Simoncelli algorithm [11]. This synthesis model measures joint wavelet statistics in the sample texture that capture correlations across position, orientation, and scale and adjusts a noise image to have the same statistics as the sample. We chose this model to create synthetic textures because it has been used in a number of recent studies to characterize texture perception [16–17,22–23] and has also been used to describe the nature of peripheral vision as well [6]. For our purposes, we did not apply an eccentricity-dependent version of the P-S procedure (as done in [22]), but instead used the entire parent texture image as the basis for subsequent texture synthesis. Examining the extent to which visual ERP components are sensitive to the discrepancies between natural textures and their synthetic counterparts in the context of this model thus adds to a growing literature using this class of summary statistics as a proxy for various stages of human vision. Finally, each image was also used to create a stimulus which had both contrast negation and texture synthesis applied. An example of a single sample texture and its appearance in all four conditions used in this study can be found in Fig 1.

### Procedure

**Fig 2:**
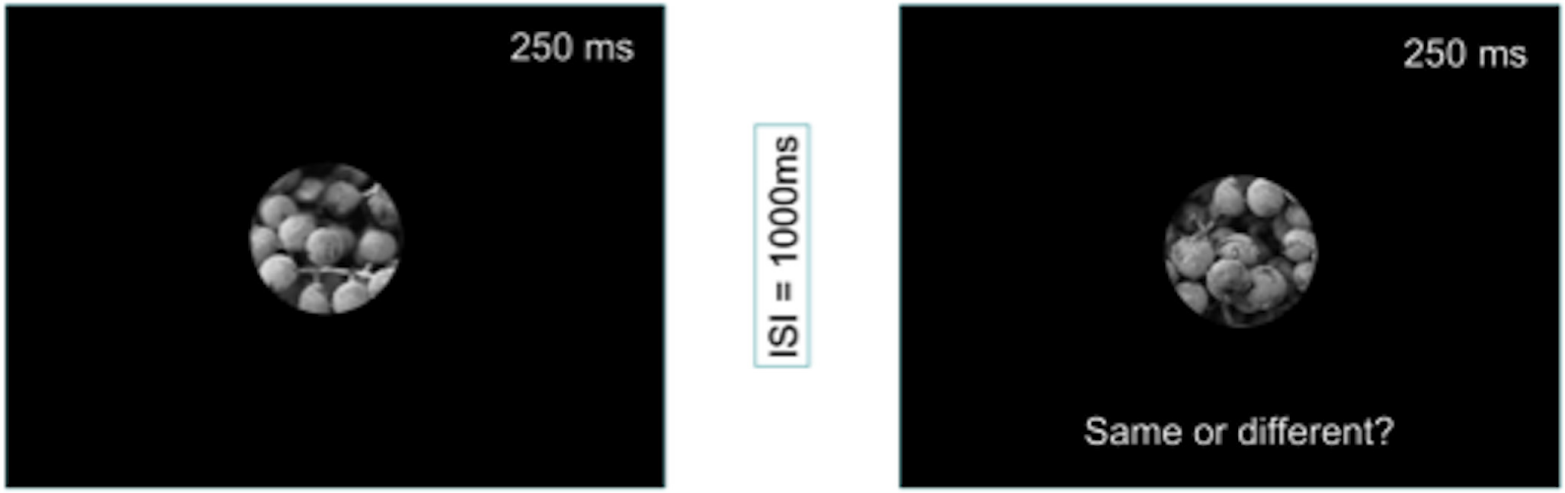
A schematic view of a single trial in our ERP task: Participants viewed sequentially presented texture patches that were either identical, or were different patches drawn from the same larger image. Contrast polarity and real/synthetic appearance were matched for the two images presented in a single trial and otherwise randomized across the session.

Participants were seated in an electrically-shielded and sound-attentuated chamber during the experiment, approximately 40cm away from the display. At this distance, our stimuli subtended a visual angle of approximately 6 degrees of visual angle and all were presented on a black background. After application of the sensor net, participants were asked to complete a same/different image matching task using the texture images described above. On each trial, we presented participants with two sequentially presented images that were either physically identical or were two unique texture patches taken from the same parent image. Each image appeared on screen for 250ms, with a 1000ms interval between the first and second image (Fig 2). Prior to image presentation, participants viewed a fixation cross for 500ms. Between trials, participants viewed a blank screen for an interval of random length bounded between 800ms and 1500ms. For each trial, participants were asked to respond “same” or “different” by pushing either “1” or “4” on a small button box they held in their lap. We note that since this task was in principle solvable using the raw intensity values of the image, we do not consider it a texture processing task *per se*, but rather a means of ensuring that participants were actively attending to the first image in each trial. The orientation of this box was flipped for half of our participants to ensure that the hand used to signal “same” was approximately balanced across participants. The two texture images appearing on each trial were always matched for appearance category—both images shared the same contrast polarity, for example, and were also matched for real/synthetic appearance. Stimulus condition varied pseudo-randomly across the entire experimental session. All stimulus timing, display parameters, and response collection routines were implemented in EPrime v2.0. Participants completed 128 trials per condition, yielding a total of 512 trials in the entire session. We note that some texture patches were presented to participants multiple times over the course of the experiment, but the specific patches that were repeated were matched across conditions.

We recorded continuous EEG at a sampling rate of 250Hz using a 64-channel Hydrocel Geodesic Sensor Net (Electrical Geodesics, Inc.), referenced to the Cz electrode. We amplified the raw signal with an EGI NetAmps 200 amplifier and applied an online band-pass filter of 0.1–100 Hz. Prior to the start of each experimental session, we established stable impedances at all sensors below a threshold of 50kΩ.

Following the experimental session, each participant’s data was analyzed using NetStation v4.3.1. (Eugene, OR). We low-pass filtered the continuous EEG data with a cut-off frequency of 30Hz, and segmented individual trials using the onset of the first stimulus presented on each trial thus avoiding any effect of participants’ responses on the observed waveform by excluding EEG data elicited by the second image entirely. For each trial, we extracted a segment that was 1000ms long, comprised of a 100ms period before stimulus onset and a 900ms period following the same. Each trial was then baseline corrected by subtracting the average value from the pre-onset period from the entire waveform.

We subsequently applied automated routines for artifact detection to label trials contaminated by eye-blinks, saccades, and bad channels. Blinks were identified using the eye electrodes positioned above and below the participant’s eyes, with a range threshold of 140 microvolts used to classify a trial as containing a blink. Saccades were similarly classified using a range threshold of 55 microvolts. Finally, bad channels were identified throughout the entire sensor array based on a range threshold of 220 microvolts. Trials containing blinks or saccades were marked and not included in subsequent averaging. Bad channels were replaced using spherical spline interpolation so that missing data could be replaced by a spatially smooth average of nearby electrodes. Following these routines for identifying and removing artifacts, we computed individual subject averages at each sensor for each condition, and re-referenced the data to an average reference. We excluded the ERP data from two participants (1 male, 1 female) due to very poor quality likely caused by excessive motion, but retained the behavioral data from these participants.

## Results

### Behavioral responses

**Fig 3:**
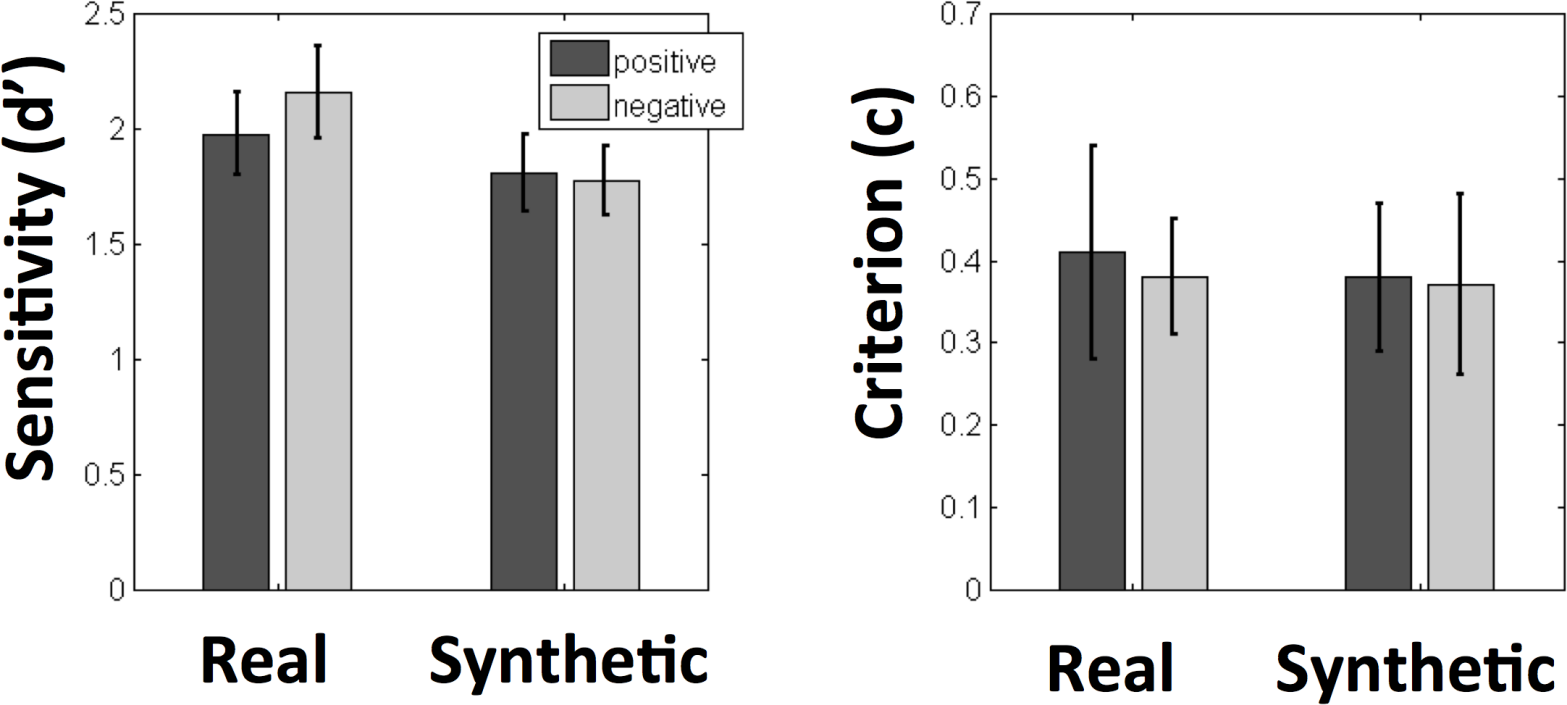
Average values for d’ and c across participants for all conditions in our experiment.

We begin by describing participants’ behavioral responses during ERP recording to estimate their ability to discriminate between texture samples in each condition (S1 File). We do not include an analysis of participants’ reaction time data here since participants were asked to wait for stimulus offset to indicate their behavioral response, which means response latency is likely not a sensitive estimate of performance in this task. For each participant, we measured their sensitivity to image-level differences between texture patches in each condition by calculating both d’ and the response criterion, c. To do so, we computed both the hit rate (correct responses of “different” to patches that were physically different) and the false alarm rate (incorrect responses of “different” to patches that were physically identical) in each condition, and obtained values for d’ and c using standard formulas. In Fig 3, we summarize the results across participants and conditions for both measures.

We analyzed these measures using a 2x2 repeated-measures ANOVA with contrast polarity (positive or negative) and real/synthetic appearance as within-subjects factors. For d’, we observed a main effect of real/synthetic appearance (F(1,16) = 6.04, p = 0.026) such that observers’ had greater sensitivity for real textures (M = 2.06) than for synthetic textures (M = 1.76). Neither the main effect of contrast polarity (F(1,16) = 1.58, p = 0.23) nor the interaction between these factors (F(1,16) = 1.95, p = 0.18) reached significance. For the response criterion c, we found no significant main effect of contrast polarity (F(1,16) = 0.32. p = 0.58) or real/synthetic appearance (F(1,16) = 0.17, p = 0.68) and the interaction between these factors also did not reach significance (F(1,16) = 0.012, p = 0.91). We conclude that sensitivity to image-level differences between patches is affected by the application of texture synthesis, but largely unaffected by contrast negation in this context.

### Event-related potentials

**Fig 4:**
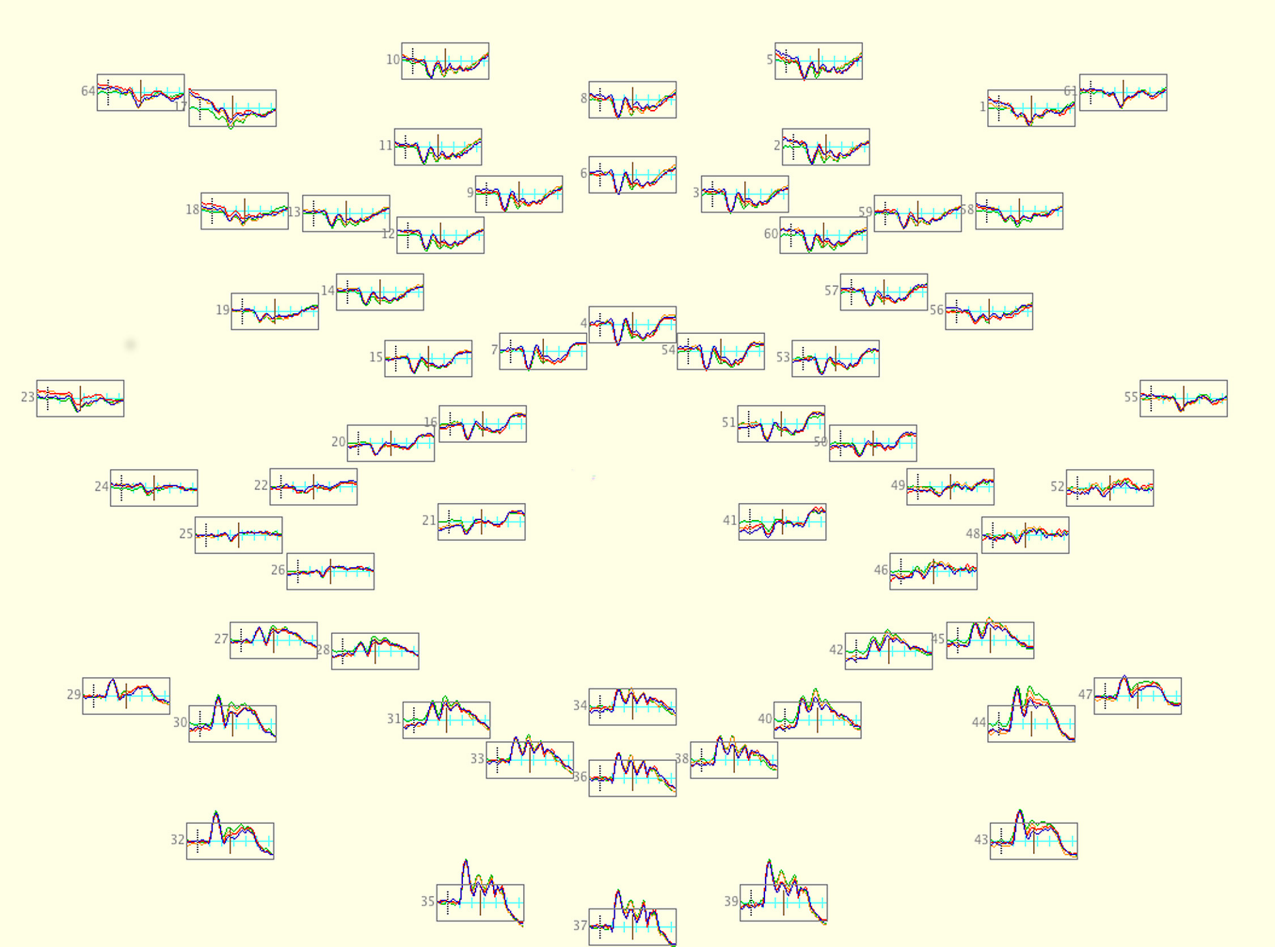
A plot displaying the ERP responses recorded in each condition across the entire scalp. Each box represents a single channel (with the exclusion of electrodes placed on the cheeks to measure eye movements and the vertex electrode) and is bounded vertically at-5μv and 5μv, horizontally at-100ms and 900ms.

**Fig 5:**
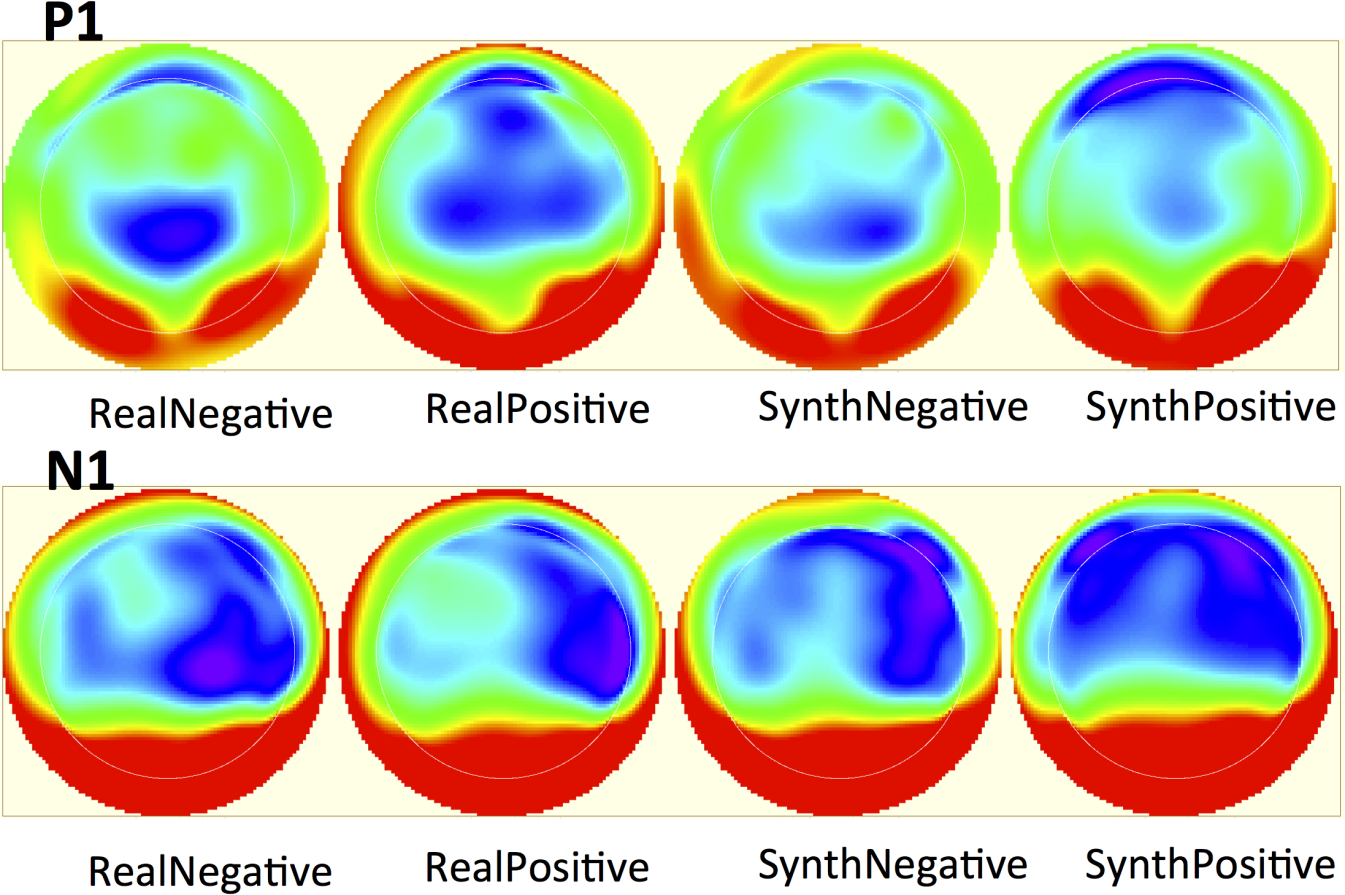
A topographic heat map of the entire scalp for both the P1 and N1 in all stimulus conditions. Warm colors signify more positive values, cool colors signify more negative values.

Since our goal was to examine the P1 and the N1, we selected three regions of interest (midline, left and right hemispheres) each comprised of sensors over the occipital-temporal portion of the scalp. Our midline region was centered at electrode Oz and contained this electrode and the three immediately adjacent electrodes (sensors 35, 36, 37, & 39 in Fig 4). The left and right regions were centered on electrodes T5 and T6 respectively, and each contained the electrodes immediately adjacent to these sensors (Left: Sensors 27, 29, 30, & 32. Right: Sensors 43, 44, 45, & 47. See Fig 4 for a map of these electrodes). Each of these three regions contained four sensors, and for each participant we averaged the data for each condition across the sensors in each region. In Fig 4, we have plotted the average ERP in each condition across the entire electrode array and in Fig 5, we have included a topographic map of both the P1 and N1 for each condition.

**Fig 6:**
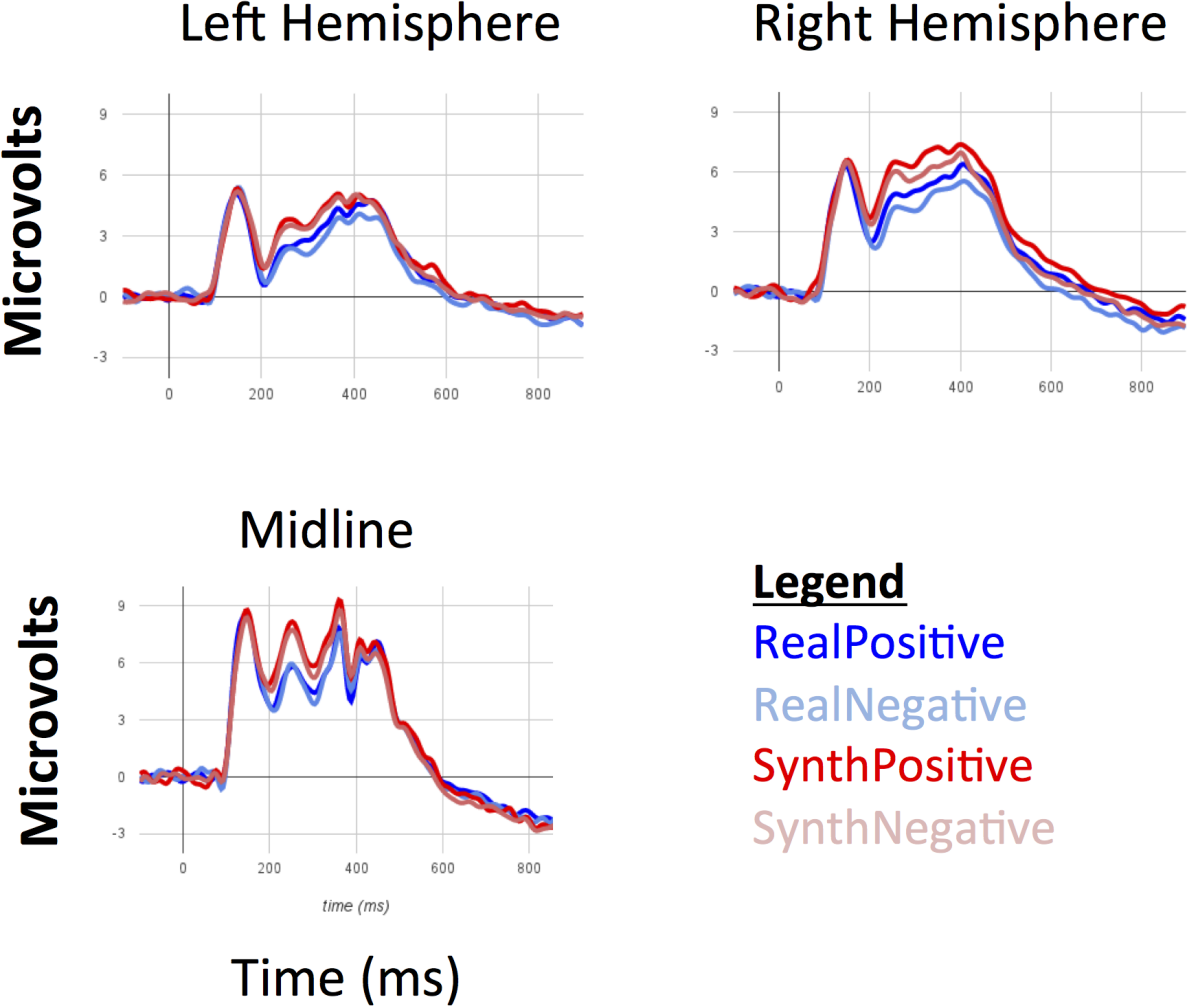
Grand average ERP waveforms for all stimulus conditions at left, midline and right occipital sites. While the P1 does not appear to be sensitive to either contrast negation or real/synthetic appearance, the N1 is sensitive to both.

All participants exhibited a clear P1/N1 complex. Using the grand average across all participants and conditions, we identified two non-overlapping time windows to describe the P1 and N1 components—the former between 115–170ms and the latter between 175–230ms. We selected these electrode sites and temporal windows based on visual inspection of the grand average waveforms (collapsed across conditions) to identify where and when the target components were most prominent. We also selected these three electrode regions so that occipital cortex was largely covered by our regions of interest. In each region, we described the component’s response to textures in each category via the mean amplitude of the ERP waveform within the relevant time window and the latency to peak response. We submitted both measures to a 2x2x3 repeated-measures ANOVA, with contrast polarity (positive/negative), real/synthetic appearance, and region (left, midline, right) as within-subject factors. Average waveforms for each stimulus condition and region calculated across all participants are depicted in Fig 6 (S2 File).

#### The P1 is not sensitive to natural appearance

This analysis revealed that the P1 component was not sensitive to any of the stimulus manipulations we implemented in this task. Neither the mean amplitude of the P1 nor its latency-to-peak were significantly impacted by contrast polarity (amplitude: F(1,14) = 0.43, p = 0.53, η^2^ = 0.03; latency-to-peak: F(1,14) = 0.002, p = 0.97, η^2^ = 0.001) or real/synthetic appearance (F(1,14) = 0.22, p = 0.65, η^2^ = 0.015 for mean amplitude, F(1,14) = 1.187, p = 0.19, η^2^ = 0.12 for latency-to-peak). We did however observe a main effect of region on the mean amplitude of the P1 (F(2,13) = 4.82, p = 0.027, η^2^ = 0.43). Pairwise comparisons between each of our three regions (Bonferroni corrected for multiple comparisons) revealed that amplitudes over midline sensors were significantly more positive than amplitudes over the left hemisphere (Mean difference = 2.9μv, s.e.m. = 0.95 μv, p = 0.028, 95% CI of the difference = [0.29 μv-5.5 μv]), but neither the difference between midline and the right hemisphere (Mean difference = 1.75 μv, s.e.m. = 0.93 μv, p = 0.24) nor the difference between right and left hemispheres (Mean difference = 1.13 μv, s.e.m. = 0.54, p = 0.17) reached significance. No such effect of region was observed for latency-to-peak, however (F(2,13) = 1.01, p = 0.39, η^2^ = 0.13) and no interactions between factors reached significance in either analysis.

#### N1 amplitude

Unlike the P1, the N1 did exhibit sensitivity to deviations from natural texture appearance. In terms of its mean amplitude, we observed significant main effects of region (F(2,13) = 4.64, p = 0.030, partial η^2^ = 0.42) and real/synthetic appearance (F(1,14) = 12.18, p = 0.004, η^2^ = 0.47). The main effect of contrast polarity did not reach significance (F(1,14) = 0.235, p = 0.64, η^2^ = 0.02). Post-hoc pairwise comparisons between the mean amplitudes measured across all three regions revealed significantly more positive amplitudes over midline sensors (M = 4.78μv, s.e.m. = 1.18) compared to left hemisphere sensors (M = 1.85μv, s.e.m. = 0.75; 95% CI of the difference in means = [0.36μv-5.5μv]), but no such difference between midline and right hemisphere sensors (Mean difference = 1.17μv, s.e.m. = 0.54μv, p = 0.15) or between left and right hemisphere sensors (Mean difference = 1.76μv, s.e.m. = 0.81μv, p = 0.14). The main effect of real/synthetic appearance was the result of more positive amplitudes for synthetic textures (M = 3.91μv, s.e.m. = 0.89) compared to real textures (M = 2.91μv, s.e.m. = 0.86; 95% CI of the difference in means = [0.39μv-1.62μv]). None of the interactions between factors reached significance.

#### N1 Latency-to-peak

The latency of the N1 component also reflects sensitivity to natural texture appearance. We observed significant main effects of real/synthetic appearance (F(1,14) = 17.39, p<0.001, η^2^ = 0.55) and contrast polarity (F(1,14) = 5.23, p = 0.038, η^2^ = 0.27). The former was the result of earlier latencies for synthetic textures (M = 196ms, s.e.m. = 2.8) compared to real textures (M = 201ms, s.e.m. = 2.9; 95% CI of the difference in means = [2.32ms-7.2ms]). The latter was the result of earlier latencies to positive contrast textures (M = 198ms, s.e.m. = 2.6) relative to negative contrast textures (M = 201ms, s.e.m. = 3.0; 95% CI of the difference in means = [0.2ms-4.1ms]). The main effect of region did not reach significance (F(2,13) = 1.69, p = 0.20, η^2^ = 0.11) and we also observed no significant interactions between factors.

We note that inspection of Fig 4 reveals other candidate differences in the ERP waveform (specifically a possible difference between 250–400ms), but since we did not have specific hypotheses regarding components in this time range, we chose not to investigate the impact of natural texture appearance on components we did not set out to analyze.

## Discussion

Our results demonstrate that visual ERPs are sensitive to the differences in appearance between natural textures and unnatural textures made by either contrast negating natural images or creating synthetic versions using the P-S algorithm. While the P1 component did not exhibit any sensitivity to the differences between these patterns, our stimuli elicited significant differences at the N1. The null results at the P1 are an important confirmation that on average, our texture stimuli did not differ systematically in low-level properties following either texture synthesis or contrast negation, since the P1 is known to be sensitive to luminance and contrast differences [31]. Texture synthesis via the P-S model generally preserves these low-level properties since the pixel intensity histogram is approximately matched in tandem with the various wavelet correlations constrained by the model during synthesis. Nonetheless, the lack of an effect at the P1 is a good indicator that low-level variability of texture appearance as a function of stimulus category was likely not the sole factor driving differential ERP responses to our different texture transformations. The P1 is also known to exhibit some sensitivity to higher-level properties of appearance, including face inversion and negation [32], meaning that this component reflects some processes that involve the measurement of image properties more complex than low-level luminance and contrast. Itier & Taylor [33] for also, suggested that a fundamental difference between the face-sensitive N170 component and the object-related N1 was that the latter mostly reflected the return to baseline following the P1 component, while the former included additional processes that were specific to face recognition. In our data, the lack of any effects at the P1 component makes it unlikely that we can explain our N1 effects with such an account. We therefore take the absence of effects at the P1 as evidence that subsequent sensitivity to natural texture statistics is not the result of artifacts of the negation or synthesis procedures applied here, suggesting that sensitivity later in the waveform reflects more complex processes.

The sensitivity to both transformations that we observed at the N1, suggest that subsequent stages of visual processing do measure sufficient statistics about texture appearance to be sensitive to the difference between natural textures and the unnatural counterparts we consider here. What processes does the N1 component reflect that may be relevant here? The N1 component reflects visual discrimination processes [34] and is also known to be larger in amplitude as a function of attention [35]. More difficult discrimination tasks tend to lead to larger amplitudes and longer peak latencies, and some aspects of our data are consistent with this account of our results: Texture synthesis led to poorer behavioral sensitivity (lower d’ scores) and larger N1 amplitudes. However, we would also expect to find longer peak latencies to synthetic textures if discrimination difficulty was the primary explanation for our results, and we observed exactly the opposite. Task difficulty thus does not offer a satisfying explanation of our peak latency data since it does not account for either the earlier latencies observed for synthetic textures (which were harder to discriminate than real ones) nor for the earlier latencies observed for positive-contrast textures (which were not easier to discriminate than negative-contrast ones).

We offer two candidate accounts (that are not mutually exclusive) of our N1 results that we think offer a more satisfying explanation of the data. First, we suggest that our data may be consistent with recent results describing sensitivity to material categories in the visual system [36]. In their recent paper, Jacobs, Baumgarner, and Gegenfurter report that they are able to classify the material category of texture images using ERP data collected within the first 200ms following stimulus onset. That is, by applying a pattern classifier to the ERP signal measured across the entire scalp, there is sufficient information to classify material categories at above-chance levels, suggesting that portions of the visual ERP signal that overlap with our P1 and N1 components carry data for material classification. Other recent results suggest that the visual processing of materials appears to transition from image-based to perceptually-based representations along the ventral visual pathway [37] such that initial visual processing is more sensitive to changes in appearance that preserve material category, while later stages achieve more invariance to these sources of variation. In both cases, the early visual system appears to carry useful information for assigning material categories (e.g., wood, water, metal) to texture patterns, and we suggest that texture synthesis and contrast negation may have different effects on N1 latency in part because these transformations may disrupt material perception in different ways. For example, synthetic textures appear to retain enough natural structure for some material properties to be perceived, including gloss [38] and intuitive categories like metal, wood, and stone [39] By comparison, contrast negation disrupts shape-from-shading [40] and also appears to disrupt the perception of translucency and gloss [41] While there is not enough data describing the critical features necessary for various material categorization tasks for us to draw this conclusion unequivocally, we suggest that our results may thus be an indication of how material perception is enacted in early vision and how it can be disrupted by manipulating natural appearance in distinct ways. Second, we note that the visual N1 is known to be sensitive to the presence of closed contours, even when those contours are only signaled by the presence of inducing elements as in a Kanisza square [42]. Given that these contours are disrupted in texture synthesis, but not contrast polarity, one account of our results is that N1 amplitude may be determined largely by their presence or absence, and the differential effects on peak latency may result from interactions between contrast polarity and perceived border ownership. We say that these two accounts are not mutually exclusive because the absence of these contours in synthetic images may be a key factor in the disruption of material perception we have described above. One way to differentiate between these two accounts could be the examination of N1 responses to textures from material categories that generally lack closed contours and proto-objects (e.g. sandy textures), since synthesis procedures would still disrupt higher-order statistics but lead to synthetic images that did not differ from the original textures in terms of contours.

Overall, our data suggest that the human visual system is indeed sensitive to the statistics of natural textures and that this sensitivity is evident at the visual N1. The particular transformations of natural appearance that we used largely preclude interpretation of our results based on sensitivity to natural contrast statistics or expertise effects, suggesting that our effects may be the result of some aspects of higher-level processing (possibly material perception or the perception of closed contours). Further work examining material categories systematically would shed important light onto the representations available to the processes indexed by the N1, and continued investigation of the critical features and visual processes necessary for various texture and material perception tasks would also help reveal what properties of natural textures are available at different stages of visual processing and what natural modes of stimulus appearance the visual system is tuned to at these levels of processing.

## Supporting Information

S1 File This supplementary file contains the raw behavioral responses recorded during the ERP session. (ZIP)

S2 File This supplementary file contains a NetStation. gmi file that contains all participants’ averaged and re-referenced ERPs in all conditions. (ZIP)
